# Supplementary material for: Involvement of a Basic Helix-Loop-Helix Gene BHLHE40 in Specification of Chicken Retinal Pigment Epithelium
Source: J Dev Biol. 2022 Oct 29;10(4):45. doi: 10.3390/jdb10040045 (PMC9680343; doi:10.3390/jdb10040045)
Supplement: Supplementary file 1 [file jdb-10-00045-s001.zip › jdb-1869185-supplementary.pdf]

Supplementary Materials

Involvement of a Basic Helix-Loop-Helix Gene *BHLHE40* in Specification of Chicken Retinal Pigment Epithelium

Toshiki Kinuhata [1,†], Keita Sato [2,†], Tetsuya Bando [2], Taro Mito [3], Satoru Miyaishi [4], Tsutomu Nohno [5] and Hideyo Ohuchi [2,*]

1 Department of Cytology and Histology, Okayama University Graduate School of Medicine, Dentistry and Pharmaceutical Sciences, 2-5-1 Shikata-cho, Kita-ku, Okayama 700-8558, Japan

2 Department of Cytology and Histology, Okayama University Faculty of Medicine, Dentistry and Pharmaceutical Sciences, 2-5-1 Shikata-cho, Kita-ku, 2 Okayama 700-8558, Japan

3 Bio-Innovation Research Center, Tokushima University, 2272-2 Ishii, Ishii-cho, Myozai-gun, Tokushima 779-3233, Japan

4 Department of Legal Medicine, Okayama University Faculty of Medicine, Dentistry and Pharmaceutical Sciences, 2-5-1 Shikata-cho, Kita-ku, Okayama 700-8558, Japan

5 Department of Cytology and Histology, Okayama University Medical School, 2-5-1 Shikata-cho, Kita-ku, Okayama 700-8558, Japan

* Correspondence: ohuchi-hideyo@okayama-u.ac.jp

† These authors contributed equally to this work.

Figure S1

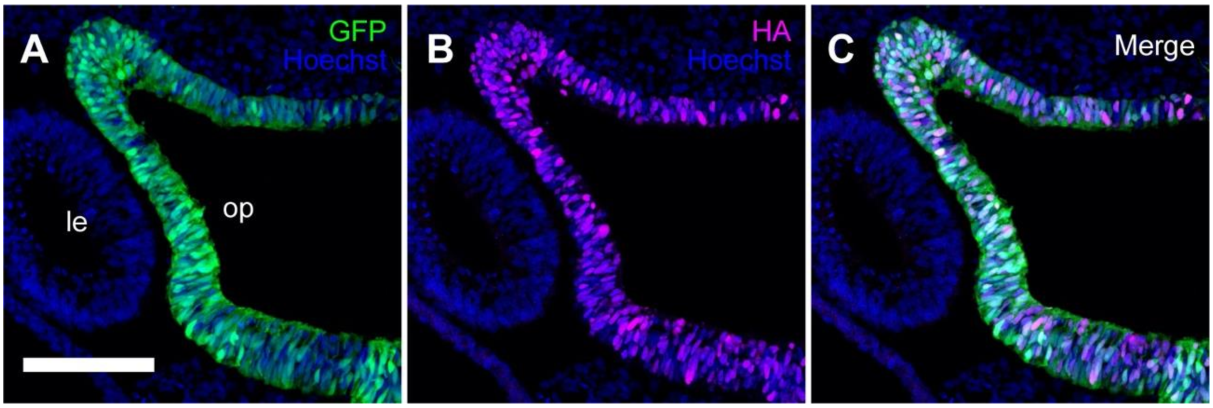

Figure S1 (data related to Figures 2 and 3): In the optic cup after *BHLHE40-HA* overexpression (from embryo #17, same as shown in Figure 3I-N), co-electroporated GFP fluorescence (**A**) is overlapped with BHLHE40-HA protein localization (**B**). Merged image of (**A**) and (**B**) is shown in (**C**). Cell nuclei were stained with Hoechst 33342. Shown images are maximum intensity projections of confocal z-series. le, lens vesicle; op, optic cup. Scale bar: 100 μm in (**A**) for (**A-C**).

Figure S2

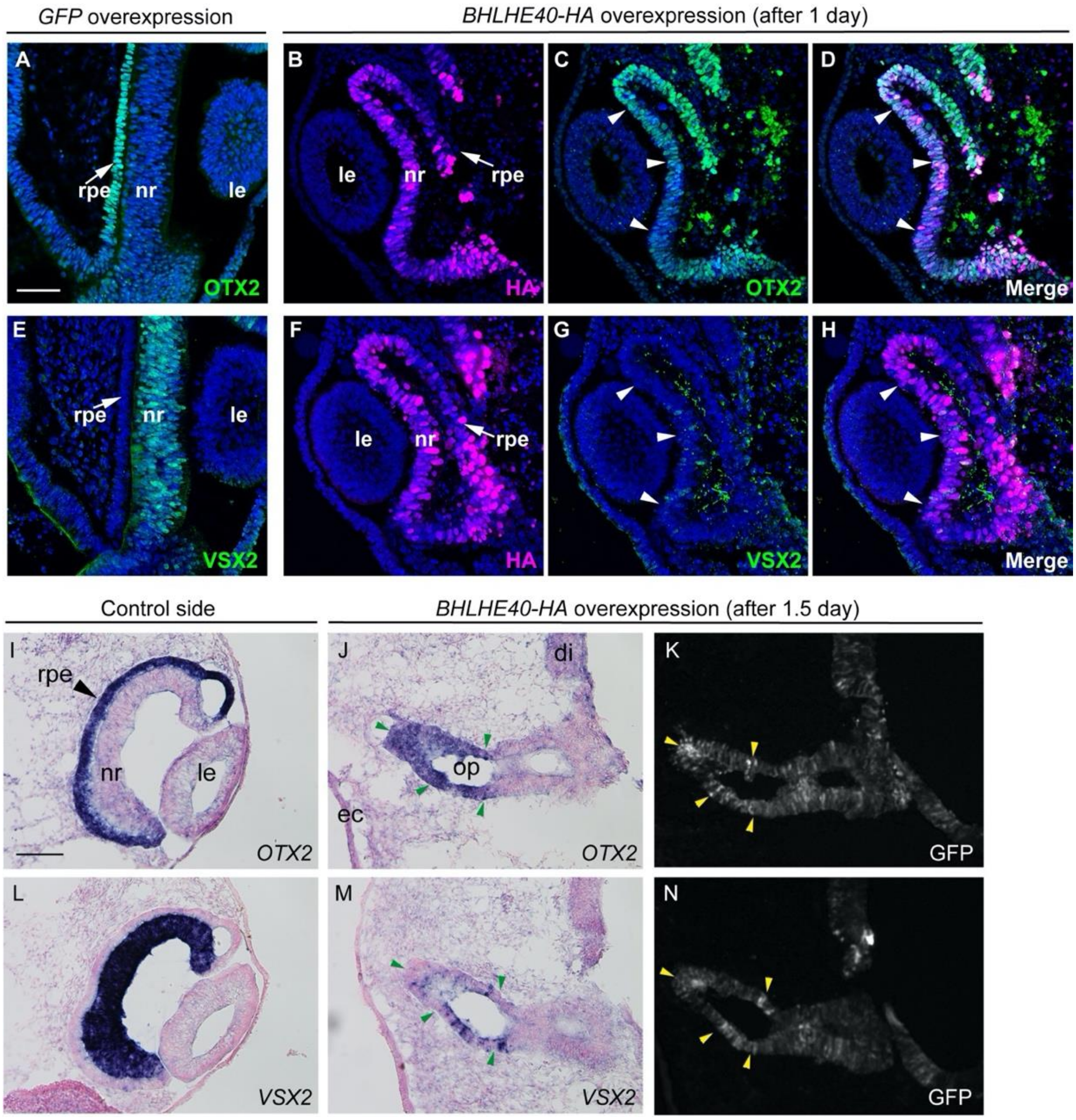

Figure S2 (data related to Figure 3): Similar data of Figure 3 from other embryos, except for those after GFP expression (**A, E**), which are identical images as shown in Figure 3A and E. Frontal sections of the optic cup from three embryos (*GFP*#2, *BHLHE40*#11, *BHLHE40*#22) are shown. Dorsal is to the up. (**A-H**) Optic cups at 1 day post electroporation (for *GFP* alone and HA-tagged *BHLHE40* overexpression). In embryo #11 after *BHLHE40* overexpression, OTX2 protein is localized to the inner layer of the OC (arrowheads in **C, D**), where VSX2 protein is not detected (arrowheads in **G, H**). Cell nuclei were stained with Hoechst 33342. Shown images are maximum intensity projections of confocal z-series. (**I-N**) Optic cups at one and half day post electroporation (HA-tagged *BHLHE40* overexpression). (**I, L**) Optic cups on the control side are shown. In (**K, N**), co-electroporated *GFP*, showing the domain of *BHLHE40* overexpression. (**J, M**) In embryo #22, optic cup development is disrupted and there is no lens formation after *BHLHE40* overexpression. *OTX2* is expressed in the distal portion of the optic cup (**J**), where *BHLHE40* is ectopically expressed (arrowheads in **J, K**). In the same domain, *VSX2* expression is downregulated but remains in some cells (arrowheads in **M, N**). Cell nuclei were stained with nuclear fast red. di, diencephalon; ec, surface ectoderm; le, lens vesicle; nr, neural retina; op, optic cup or deformed optic vesicle in (**J-N**); rpe, developing RPE. Scale bar: 50 μm in (**A**) for (**A-H**), 100 μm in (**I**) for (**I-N**).

Figure S3

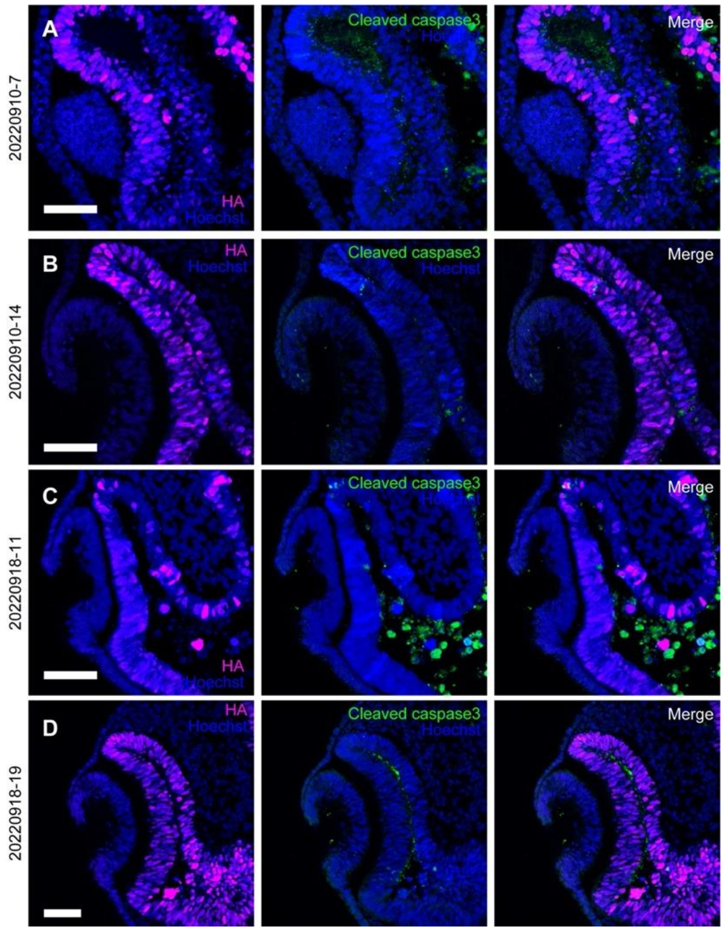

Figure S3: ***BHLHE40-HA*** **expressing cells do not show cell death as revealed by immunofluorescence using anti-cleaved caspase 3.** In four embryos examined (**A-D**) (embryo identification numbers are shown on the leftmost), none of the HA-immunoreactive cells are positive for cleaved caspase 3. Cell nuclei were stained with Hoechst 33342. Shown images are maximum intensity projections of confocal z-series. Scale bar: 50 μm in (**A-D**) for all images.

Figure S4

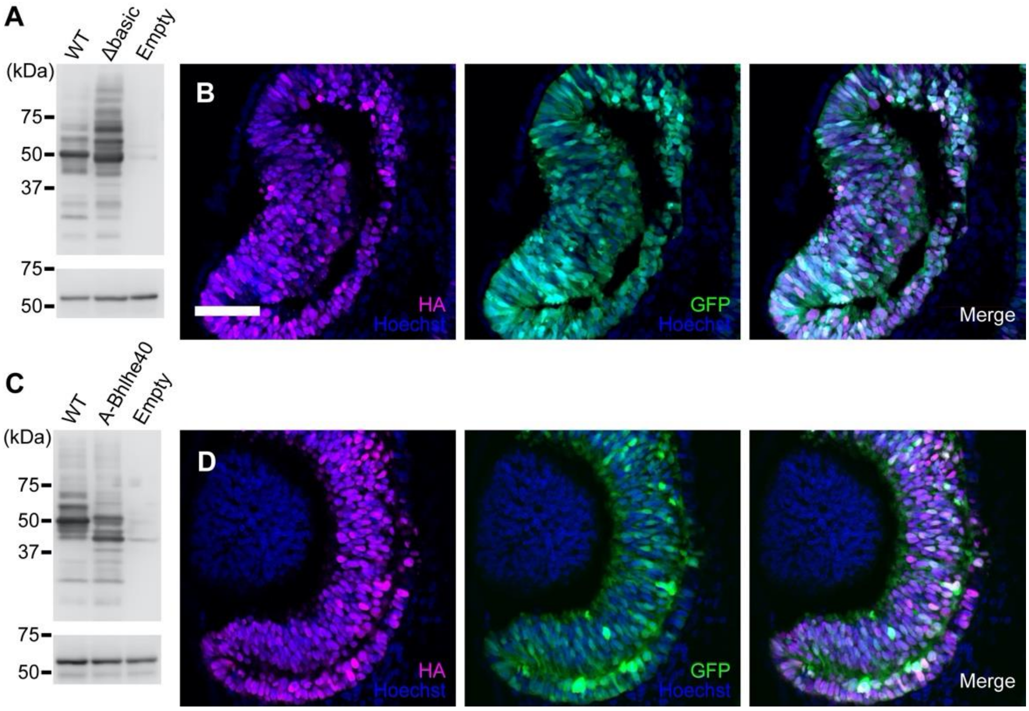

Figure S4: Validation of expression of HA-tagged presumed dominant negative forms of BHLHE40. (**A, C**) Western bot analysis of HEK293T cells transfected with chicken Δbasic *BHLHE40*-HA/pCAGGS (**A**) or Δbasic with acidic extension *BHLHE40*-HA (**C**) vectors. Alpha tubulin from the same samples was detected for loading reference. (**B, D**) Confocal microscopic images show that HA-tagged presumed dominant negative forms of BHLHE40 are detected all over the optic cup by immunofluorescence using the anti-HA antibody. Secondary antibody to anti-HA was Alexa Fluor 568-conjugated anti-rat IgG. Co-electroporated GFP and merged images are shown in the middle and right panels, respectively. Cell nuclei were stained with Hoechst 33342. Shown confocal images are maximum intensity projections of z-series. Scale bar: 50 μm in (**B**) for all confocal images.

Figure S5

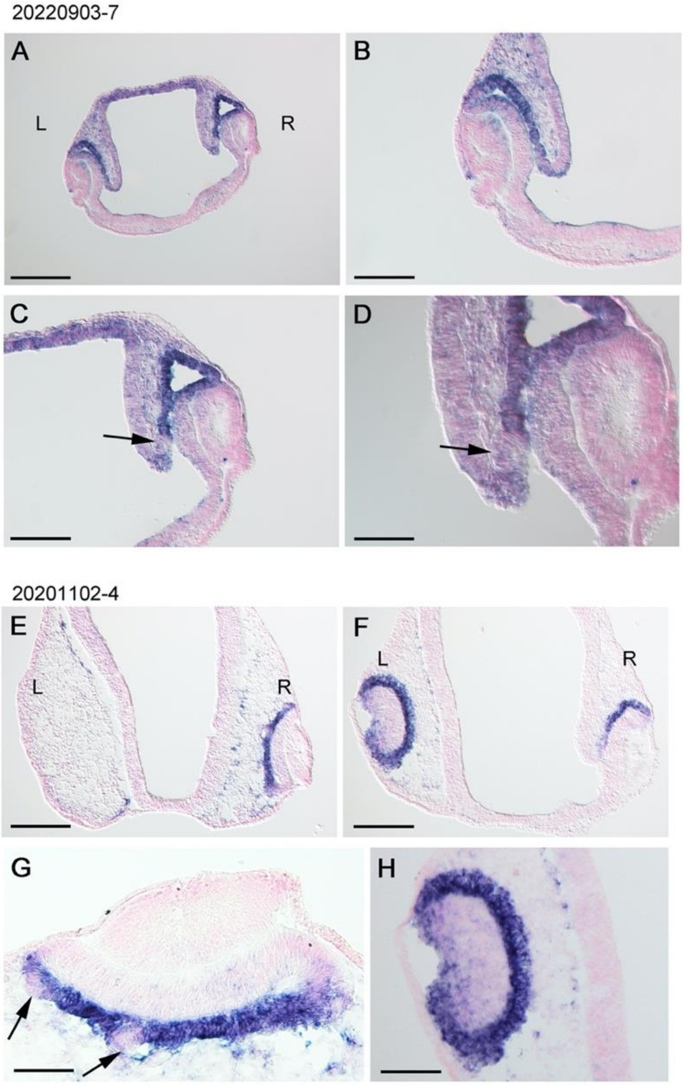

Figure S5 (data related to Figure 4): In situ hybridization (ISH) on the frontal section of the chicken embryonic head after 1 day post electroporation (embryo #7 for **A-D** and #4 for **E-H**). Enlarged view of the optic cup (OC) in (**A**) on the left (L) control side is shown in (**B**), and that of the right (R) *Lhx1*-overexpressed one is shown in (**C, D**). *BHLHE40* expression is downregulated in a portion of the outer layer of the OC (arrows in **C, D**). In embryo #4, the head sections were cut oblique and the left control OC is not seen in (**A**). Enlarged view of the right OC is shown in (**G**) and that of the left control OC is shown in (**H**). *BHLHE40* expression is downregulated in two portions of the outer layer of the OC (arrows in **G**). Cell nuclei were stained with nuclear fast red. Scale bars: 200 µm (**A, E, F**), 100 µm (**B, C, H)**, 70 µm (**G**), 50 µm (**D**).

Figure S6

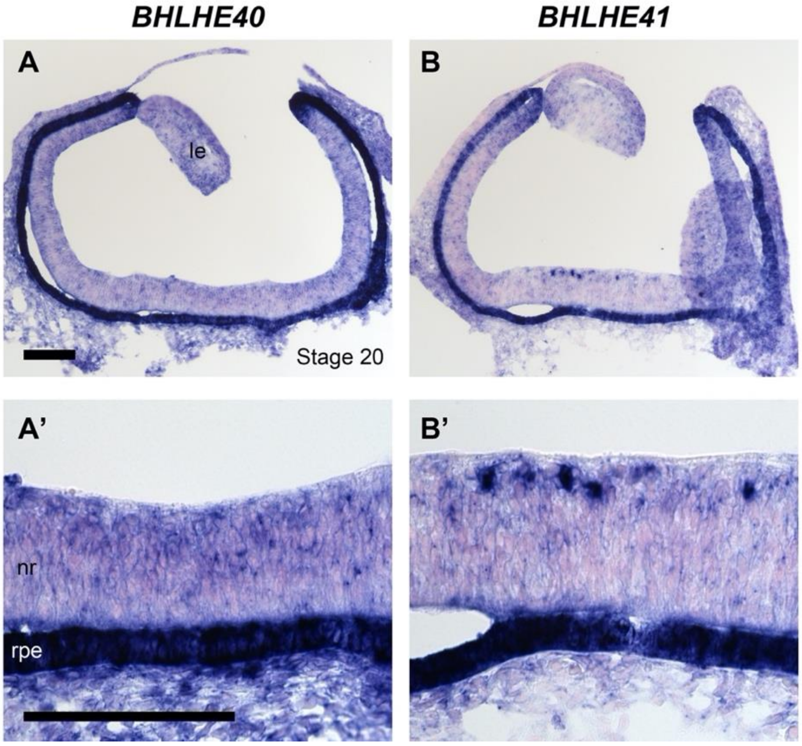

Figure S6: Expression patterns of *BHLHE40* (A,A′) and *BHLHE41* (B,B′) in the developing chicken eye at stage 20. *BHLHE41* is expressed by developing retinal ganglion cells at the central retina in addition to the developing RPE and ciliary marginal zone. le, lens vesicle; nr, neural retina; rpe, developing RPE. Scale bars: 100 μm.

Figure S7

```
                       10         20         30         40         50         60         70         80         90        100
               ....|....| ....|....| ....|....| ....|....| ....|....| ....|....| ....|....| ....|....| ....|....| ....|....|
CHICK BHLHE40  ATGGAGCGCA TCCCCAGCGC GCAGCCCCCG CCCGTGTGCC TGGGCAAGCT GCCCGCCCTG GAG-AGCGCC GATGTGCCGG GGCTGGACTT CGCG-CACAT
CHICK BHLHE41  ATGGA-CG-- -----AG-G- GCATCTCCCG CC----TGCC CGAGCG-GCA GCT-GC--TG GAGCACCGC- GATTTCCTAG GGCTGGAGTA CCCGGCGC-T

                      110        120        130        140        150        160        170        180        190        200
               ....|....| ....|....| ....|....| ....|....| ....|....| ....|....| ....|....| ....|....| ....|....| ....|....|
CHICK BHLHE40  GTACCAAGTG TACAAGCCCA GGAGGGGTT AAAGAGGAGC GAGGACAGCA AGGACACCTA CAAGCTGCCC CACAGGCTGA TAGAGAAGAA GCGGCGCGAT
CHICK BHLHE41  CTAC-ATGTG --CAAGCCCA AGCGCGGCGT GAAGCGGGAC GAG---AGCA AGGAGACGTA CAAACTGCCG CACCGGCTGA TCGAGAAGAA GCGGCGGGAC

                      210        220        230        240        250        260        270        280        290        300
               ....|....| ....|....| ....|....| ....|....| ....|....| ....|....| ....|....| ....|....| ....|....| ....|....|
CHICK BHLHE40  AGGATCAACG AGTGCATCGC CCAGCTCAAG GACCTGCTGC CCGAGCACCT CAAGCTGACG ACCCTGGGCC ACCTGGAGAA GGCGGTGGTG CTGGAGCTGA
CHICK BHLHE41  AGGATCAACG AGTGCATCGC GCAGCTGAAG GACCTGCTGC CCGAGCACCT CAAGCTCACG ACGCTGGGGC ACCTGGAGAA GGCGGTGGTG CTGGAGCTGA

                      310        320        330        340        350        360        370        380        390        400
               ....|....| ....|....| ....|....| ....|....| ....|....| ....|....| ....|....| ....|....| ....|....| ....|....|
CHICK BHLHE40  CCCTGAAGCA CGTGAAGGCG CTGACGGCGC TCATCGAGCA GCAGCAGCAG AAGATCCTGG CGCTGCAGAG CGGGCTGCAG GCGGGTGACC TGTCAGCGAG
CHICK BHLHE41  CGCTGAAGCA CCTGAAGGCG CTGACGGCGC TGACGGAGCA GCAGCACCAG AAGATCGTCG CGCTGCAGAG CGGGGAGCGC TCCG-TCAAG TCTCCG-GTG

                      410        420        430        440        450        460        470        480        490        500
               ....|....| ....|....| ....|....| ....|....| ....|....| ....|....| ....|....| ....|....| ....|....| ....|....|
CHICK BHLHE40  GAGCCTCGAC TCCAGCCAGG A-GATGTTCC GCTCCGGCTT CCAGCTGTGC GCCAAGGAGC TGCTGCAGTA TGTGGCC--- ----A-AGCA CGACAACAAG
CHICK BHLHE41  CAGGC-CGAC ------CTGG ACGCT-TTCC ACTCGGGCTT TCAGACGTGC GCCAAGGAGG TGCTGCAGTT CCTGTCCCGC TCCGAGAGCT GGACCCCCCG

                      510        520        530        540        550        560        570        580        590        600
               ....|....| ....|....| ....|....| ....|....| ....|....| ....|....| ....|....| ....|....| ....|....| ....|....|
CHICK BHLHE40  -GAGCTGAAG GCAGCGC--A GCTGGTGGGG CACCTGCACC GCGTGGCGGC CGAGCTGCTG CCGGGC---- GGCCCCAA-- ---------G GACCTGAAGG
CHICK BHLHE41  CGAGCAGC-- GCTGCGCCCA GCTCCTCGGC CACCTGCACG CCGTCTCCTC GCAGTTCCTG CCCGGCCGC GGCTCCTCTC GCCGCCGCCG GGCCCGCTCG

                      610        620        630        640        650        660        670        680        690        700
               ....|....| ....|....| ....|....| ....|....| ....|....| ....|....| ....|....| ....|....| ....|....| ....|....|
CHICK BHLHE40  AGAAGG---- ---------- ---------- ------CGGT ------GCCG GGGGGCAAAG CGGGCGAGGG GCGCGGGCAG AACTGCGTGC CCGTCATCCA
CHICK BHLHE41  CCAAGGGACC CTCCTCCTCT TCCTCCCCGC CCGCCCCGCT CCGCGCGCCG GCCCGCAA-G CCGG--AGGG -C-CAGGC-G CACTGCGTGC CCGTCATCCA

                      710        720        730        740        750        760        770        780        790        800
               ....|....| ....|....| ....|....| ....|....| ....|....| ....|....| ....|....| ....|....| ....|....| ....|....|
CHICK BHLHE40  AAGGACATTC GCTGCCTCC- AGCGGGGAGC AGAGCGGCAG CGACACAGAC ACGGACAGCG GCTACGGGGG GGAGCTGGAG A--------- AAAGCGACTC
CHICK BHLHE41  GCGGAC--TC AC-GCCGCCG AGCCGGGCGC CGAG----AC CGACACGGAC ACGGACAGCG GCTACGGCGG CGAGGCCGAG GCGCGGCCGG AGCGCGGCGC

                      810        820        830        840        850        860        870        880        890        900
               ....|....| ....|....| ....|....| ....|....| ....|....| ....|....| ....|....| ....|....| ....|....| ....|....|
CHICK BHLHE40  CAAACCGGAG CAGCCCTATT TCCCAAAGGA TACCGAACTG AAATACGCCG TCCAGG-A-G AGGATCAGCT C-CATC-AAG CAG--GAG-- ---AGCGAGG
CHICK BHLHE41  GGGGCCGGCG GGGCCGC--- TCCCCGCGCT GCCCGTCAAG CAGGA-GCCG GCGGGGACG AGGCGCCGCC CGCGCCCAAG CGGCCGAGGC TGGAGCGGGG

                      910        920        930        940        950        960        970        980        990       1000
               ....|....| ....|....| ....|....| ....|....| ....|....| ....|....| ....|....| ....|....| ....|....| ....|....|
CHICK BHLHE40  ----ACCCTC CAGCCAAGAG GAGCA--GGC TGGAGGTGCC CGATGAT--G ACAGCCCCTT TGGCG-GCGA GGTGATGG-G CAC--CGCGG GCGGCTTCCT
CHICK BHLHE41  CGGCAGCCCC CCGCCCGGCG CCGCCCTGCC CGGCGCTGCC CGCGGCGCCG AC-GCCGCGC TGCTGAGCTC GCTGATGGCG CTGGGCGCGG GCGGCGGCGC

                     1010       1020       1030       1040       1050       1060       1070       1080       1090       1100
               ....|....| ....|....| ....|....| ....|....| ....|....| ....|....| ....|....| ....|....| ....|....| ....|....|
CHICK BHLHE40  GCCCCCCCAC GCCCA-CCAG C--CCCCCTT GTGCCTGCCC TTCTACCTGA TCCCACCGTC ---CGCCACC GCCTACCTGC ---CCATGCT GGAGAAGTGC
CHICK BHLHE41  GGCGCCCTTC GGACAGCCGG CGGCCCCCTT CTGCCTGCCC TTCTACTTCA TCTCCCCCTC GGCCGCCGCC GCCTACGTGC AGCCCTTCCT GGATAAGGGC

                     1110       1120       1130       1140       1150       1160       1170       1180       1190       1200
               ....|....| ....|....| ....|....| ....|....| ....|....| ....|....| ....|....| ....|....| ....|....| ....|....|
CHICK BHLHE40  ----TGG--- --------TA CCCGGCCTCC GTC--CCCG- TGCTGTACCC CGGGCTGCCC GCGCCTGCCG CTGC-GCTCA CCAGCTTTGT GGGCCCCGAC
CHICK BHLHE41  GGCCTGGAGA AGTACCTCTA CCCCGCCGCC CCCATCCCGC TGCTCTACCC CGGCATCCCG GCGCAGGCGG CCGCCGC-CG CC-GCCGCGG CCGCCGCGGC

                     1210       1220       1230       1240       1250       1260       1270       1280       1290       1300
               ....|....| ....|....| ....|....| ....|....| ....|....| ....|....| ....|....| ....|....| ....|....| ....|....|
CHICK BHLHE40  AAGCTGTCCC C--CCCACTG C--------- -TGCT--GCC CC-----AAA GACTGCC--- CT--CCCCGG GGCC-----T GCCCGCTCCC CCATCGACTC
CHICK BHLHE41  C-GCCGCCTC CTTCCC-CTG CCTCTCGTCC GTGCTCGGCC CCGCCGAAAA GGCGGCCGGG CTGTCCCCGG CGCCCCACCT GCC-GC-CCC CCTTCGCCGC

                     1310       1320       1330       1340       1350       1360       1370       1380
               ....|....| ....|....| ....|....| ....|....| ....|....| ....|....| ....|....| ....|....| ....|
CHICK BHLHE40  CTCGGCC--- -------CTG CTGC--AG-- --GCGCTGAA GCAGATCCC- ---CC--CGT TGAACTTGGA AACCAAAGAC --TAA
CHICK BHLHE41  CGCCGCCGCC GCGGTGCCCG CCGCCGAGCC CGGCGAGGAA GCGGAGCCCG CGGCCGCCGA GGAGCCCGGC GCCGAAGGGC CGTGA
```

Figure S7: Alignment of coding DNA sequences for chicken BHLHE40 and BHLHE41. The nucleotide identity is 63%. Identical nucleotides are highlighted in yellow.

Figure S8

```
                       10         20         30         40         50         60         70         80         90        100
               ....|....| ....|....| ....|....| ....|....| ....|....| ....|....| ....|....| ....|....| ....|....| ....|....|
CHICK BHLHE40  M-ERIPSAQP PPVCLGKLPA LESADVPGLD FAHMYQVYKP RRGLKRSEDS KDTYKLPHRL IEKKRRDRIN ECIAQLKDLL PEHLKLTTLG H--LEKAVVL
HUMAN BHLHE40  M-ERIPSAQP PPACLPKAPG LEHGDLPGMY PAHMYQVYKS RRGIKRSEDS KETYKLPHRL IEKKRRDRIN ECIAQLKDLL PEHLKLTTLG H--LEKAVVL
MOUSE BHLHE40  M-ERIPSAQP PPTCLPKAPG LEHGDLSGMD FAHMYQVYKS RRGIKRSEDS KETYKLPHRL IEKKRRDRIN ECIAQLKDLL PEHLKLTTLG H--LEKAVVL
DANRE BHLHE40  M-ERITSAQP PP-CMSKHPS LDISDMQGMD FP-MY-VYKP RRGMKRSEDS KDTYKLPHRL IEKKRRDRIN ECIAQLKDLL PEHLKLTTLG H--LEKAVVL
CHICK BHLHE41  MDEGISRLPE RQ-------L LEHRDFLGLE YPALY-MCKP KRGVKR-DES KETYKLPHRL IEKKRRDRIN ECIAQLKDLL PEHLKLTTLG H--LEKAVVL
HUMAN BHLHE41  MDEGIPHLQE RQ-------L LEHRDFIGLD YSSLY-MCKP KRSMKR-DDT KDTYKLPHRL IEKKRRDRIN ECIAQLKDLL PEHLKLTTLG H--LEKAVVL
MOUSE BHLHE41  MDEGIPHLQE RQ-------L LEHRDFIGLD YSSLY-MCKP KRSLKR-DDT KDTYKLPHRL IEKKRRDRIN ECIAQLKDLL PEHLKLTTLG H--LEKAVVL
DANRE BHLHE41  MEERISRMQN RQ-------F LDHADFLGVE YSSLY-MCKS KRGMKR-EEG KDAYKLPHRL IEKKRRDRIN ECIGQLKDLL PEHLKLTTLG H--LEKAVVL
                                                                        basic helix-loop-helix (bHLH) domain
                      110        120        130        140        150        160        170        180        190        200
               ....|....| ....|....| ....|....| ....|....| ....|....| ....|....| ....|....| ....|....| ....|....| ....|....|
CHICK BHLHE40  ELTLKHVKAL TALIEQQQQK ILALQSGLQA GDLSARSLDS SQEMFRSGFQ LCAKELLQYV AKHD--N-KE LKAAQLVGHL HRVAAELLPG G---------
HUMAN BHLHE40  ELTLKHVKAL TNLIDQQQQK IIALQSGLQA GELSGRNVET GQEMFCSGFQ TCAREVLQYL AKHE--NTRD LKSSQLVTHL HRVVSELLQG G--------T
MOUSE BHLHE40  ELTLKHVKAL TNLIDQQQQK IIALQSGLQA GDLSGRNLEA GQEMFCSGFQ TCAREVLQYL AKHE--NTRD LKSSQLVTHL HRVVSELLQG G--------A
DANRE BHLHE40  ELTLKHVKAL NNLLEQQQQK IISLQNGLQI GEQGNGPSEN SEEMFRSGFH LCAKEVLQFL ANQE--TMRD LTTAHIIEHL QKVASELIQS P--------P
CHICK BHLHE41  ELTLKHLKAL TALTEQQHQK IVALQSGERS VK---SPVQA DLDAFHSGFQ TCAKEVLQFL SRSESWTPRE QRCAQLLGHL HAVSSQFLPG PRLLSPPPGP
HUMAN BHLHE41  ELTLKHLKAL TALTEQQHQK IIALQNGERS LK---SPIQS DLDAFHSGFQ TCAKEVLQYL SRFESWTPRE PRCVQLINHL HAVATQFLPT PQLLTQQV-P
MOUSE BHLHE41  ELTLKHLKAL TALTEQQHQK IIALQNGERS LK---SPVQA DLDAFHSGFQ TCAKEVLQYL ARFESWTPRE PRCAQLVSHL HAVATQLL-T PQV------P
DANRE BHLHE41  ELTLKHLNAL TAVTEQQHQK IIALQNGERS LK---SSLQA DLDAFHSGFQ ACAKEVLQYL NKVENWTARE QRCTRLINHL HKVSAQFQPG TGILQQ----
                                                                 Orange domain
                      210        220        230        240        250        260        270        280        290        300
               ....|....| ....|....| ....|....| ....|....| ....|....| ....|....| ....|....| ....|....| ....|....| ....|....|
CHICK BHLHE40  --------PK DLKEKAVPG- --GKAGEGRG QNCVPVIQRT FAASSGEQSG SDTDTDSGYG GELEKSDSKP EQP--YFPKD TELKYAVQER ISSIKQE--S
HUMAN BHLHE40  SRKPSDPAPK VMDFKEKPSS P-AKGSEGPG KNCVPVIQRT FAHSSGEQSG SDTDTDSGYG GESEKGDLRS EQP--CFKSD HGRRFTMGER IGAIKQE--S
MOUSE BHLHE40  SRKPLDSAPK AVDLKEKPSF L-AKGSEGPG KNCVPVIQRT FAPSGGEQSG SDTDTDSGYG GELEKGDLRS EQP--YFKSD HGRRFAVGER VSTIKQE--S
DANRE BHLHE40  SPRLDEPASK AQESREKPSG LQPKAAEGHA KNCVPVIQRT YPHSS-EQSG SDTDTDSGYG GEYEKRDQKA QRPDCYVKES GALKYS---- -SSIKEE--Q
CHICK BHLHE41  LAKG----PS SSSSPPAP-L RAPARKPEGQ AHCVPVIQRT H-AAE-PGAE TDTDTDSGYG GEAEARPERG AGP------- -----AGPLP ALPVKQEPAG
HUMAN BHLHE41  LSKGTG-APS AAGSAAAPCL ERAGQKLEPL AYCVPVIQRT QPSAE-LAAE NDTDTDSGYG GEAEARPDRE KGK------- -----GAGAS RVTIKQEPPG
MOUSE BHLHE41  SGRGSGRAPC SAGAAAAS-- -----GPERV ARCVPVIQRT QPGTE---PE HDTDTDSGYG GEAEQG---- ---------- ---------- RAAVKQEPPG
DANRE BHLHE41  --------PV AGDDAPER-- ---DTQRDPQ ANCVPVIQRT Q---NLELNE NDTDTDSGYG GEAEKGDGKS EKG------- -----CDTAK GVKIKQE-FG

                      310        320        330        340        350        360        370        380        390        400
               ....|....| ....|....| ....|....| ....|....| ....|....| ....|....| ....|....| ....|....| ....|....| ....|....|
CHICK BHLHE40  EDPPAKRSRL EVPDDD---- ---------- ---------- ---------- ------SPFG GEVMGTAGGF LPPHAHQPPL CLPFYLIPPS AT-AYL-PM-
HUMAN BHLHE40  EEPPTKKNRM QLSDDE---- ---------- ---------- ---------- -----GHFTS SDLIS--SPF LGPHPHQPPF CLPFYLIPPS AT-AYL-PM-
MOUSE BHLHE40  EEPPTKKSRM QLSEEE---- ---------- ---------- ---------- -----GHFAG SDLMG--SPF LGPHPHQPPF CLPFYLIPPS AT-AYL-PM-
DANRE BHLHE40  DEPPSKRPRS DSSEDE---- ---------- ---------- ---------- ------SLSG HDVVGGHSPY VSFSPPQ-PL CMPFYLFPPG AAAAYL-PM-
CHICK BHLHE41  DEAPPAPKRP RLERGG---- ---------- -----SPPPG AALPGAARGA DAALLSSLMA LGAGGGAAPF GQP---AAPF CLPFYFISPS AAAAYVQPFL
HUMAN BHLHE41  EDSP-APKRM KLDSRGGGSG GGPGGGAAAA AAALLGPDPA AA--AALLRP DAALLSSLVA FGGGGG-APF PQPAAAAAPF CLPFCFLSPS AAAAYVQPFL
MOUSE BHLHE41  DSSP-APKRP KLEARG---- ---------- ---------- -----ALLGP EPALLGSLVA LGGG---APF AQP--AAAPF CLPFYLLSPS AA-AYVQPWL
DANRE BHLHE41  DERVTKKAKM NWSANG---- ---------- -----GSDST ST------RP DVALMNSLMG MTGVGG---- -----QQTPF CMPFYFINPS AAASYM-PLF

                      410        420        430        440        450        460        470        480        490        500
               ....|....| ....|....| ....|....| ....|....| ....|....| ....|....| ....|....| ....|....| ....|....| ....|....|
CHICK BHLHE40  ----LEKCWY PAS------- -VPVLYPGLP --------AP AAALTSFVGP DKLSPPLLLP QRLPSPGPA- ---------- ---RSPIDSS ALLQALKQIP
HUMAN BHLHE40  ----LEKCWY PTS------- -VPVLYPGLN --------AS AAALSSFMNP DKISAPLLMP QRLPSPLPA- ---------- ---HPSVDSS VLLQALKPIP
MOUSE BHLHE40  ----LEKCWY PTS------- -VPVLYPGLN --------TS AAALSSFMNP DKIPTPLLLP QRLPSPL-A- ---------- ---HSSLDSS ALLQALKQIP
DANRE BHLHE40  ----LEKCWY PGA------- -MPVLYPGLG --------SS PASLS----P EKLPSSMVMS SRVGSPVST- ---------- ---PTSMDSP ALLQALKQVP
CHICK BHLHE41  DKGGLEKYLY PAA------- PIPLLYPGIP --AQAAAAAA AAAAAAASFP CLSSVLGPA- EKAAGLSPAP HLP------- ---PPFAAAA AAVPAAEPGE
HUMAN BHLHE41  DKSGLEKYLY PAAAAA---- PFPLLYPGIP APAAAAAAAA AAAAAAAAFP CLSSVLSPPP EKAGA-AAAT LLPHEVAPLG APHPQHPHGR THLPFAGPRE
MOUSE BHLHE41  DKSGLDKYLY PAAAA----- PFPLLYPGIP --------AA AAAAAAAAFP CLSSVLSPPP EKAGATAGAP FLAHEVAPPG PLRPQHAHSR THLPRA----
DANRE BHLHE41  DKSHLEKLVY PAAAAAALTT PFPWLYPGIP ------THAS AAAAAAIAFP NAST------ DKTSGFDAAS SKDDE----- ---PESPDDD LSNEADLASP
                                                         alanine/glycine-rich region
                      510        520
               ....|....| ....|....| ...
CHICK BHLHE40  PLNLETKD-- ---------- ---
HUMAN BHLHE40  PLNLETKD-- ---------- ---
MOUSE BHLHE40  PLNLETKD-- ---------- ---
DANRE BHLHE40  PLNLETKD-- ---------- ---
CHICK BHLHE41  EAEPAAAE-- ---EPGA--- EGP
HUMAN BHLHE41  PGNPESSAQE DPSQPGK--- EAP
MOUSE BHLHE41  -VNPESSQ-E DATQPAK--- DAP
DANRE BHLHE41  ASEDHGSEID TSHQPQRNDN DGT
```

Figure S8: Alignment of BHLHE40 and BHLHE41 proteins of chicken, human, mouse and zebrafish. bHLH and orange domains, and alanine and glycine-rich region in the C-terminus are shown. Gene IDs for referenced amino acid sequences are as follows: CHICK BHLHE40 (416108), HUMAN BHLHE40 (8553), MOUSE BHLHE40 (20893), DANRE BHLHE40 (324413), CHICK BHLHE41 (101750689), HUMAN BHLHE41 (79365), MOUSE BHLHE41 (79362), DANRE BHLHE41 (563771).

Figure S9

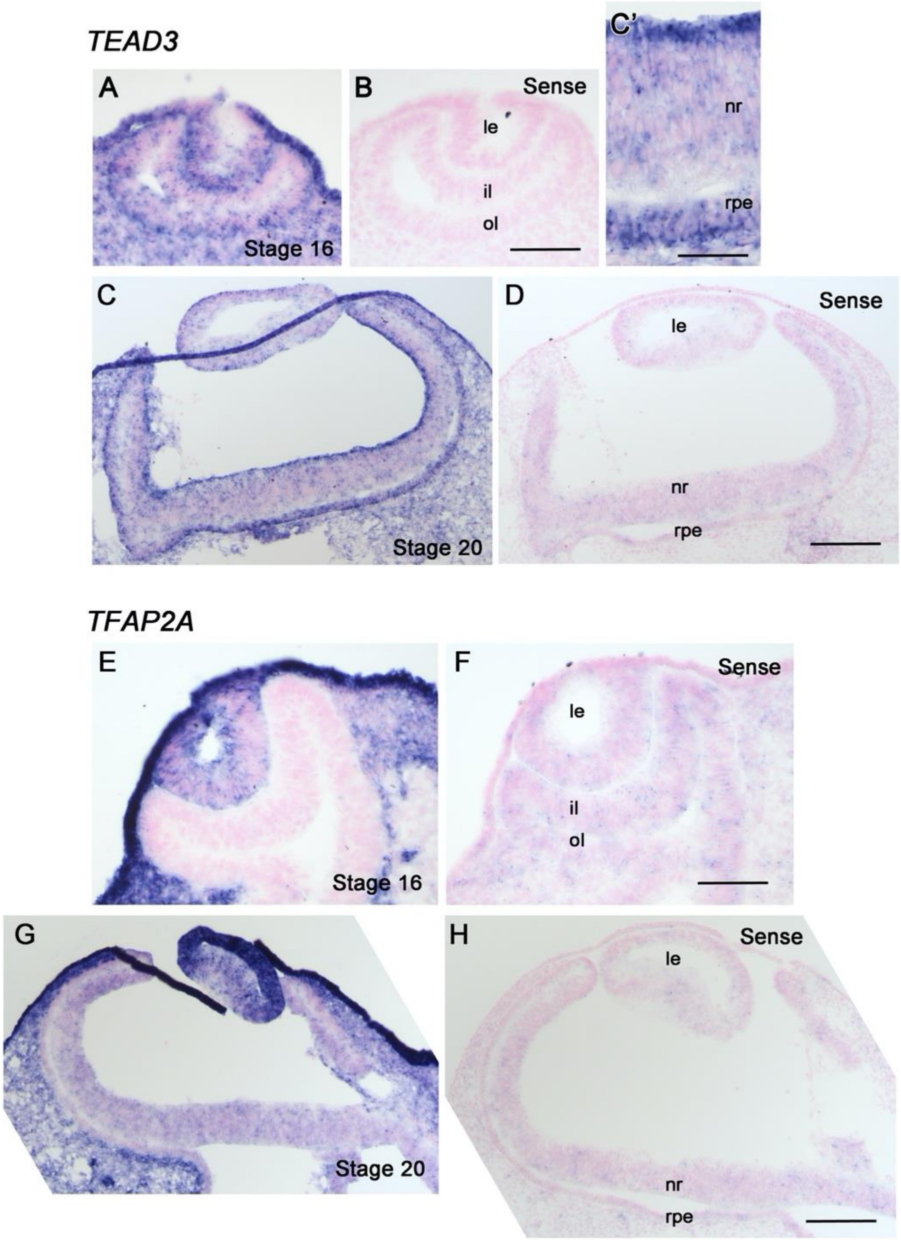

Figure S9: **Expression patterns of *TEAD3* and *TFAP2A* in the developing chicken eye.** (**A-D**) *TEAD3* is expressed in the surface ectoderm (SE) and developing eye at stages 16 (**A,B**) and 20 (**C,C′,D**). (**C′**) Enlarged view of the developing retina is shown. *TEAD3* mRNA signals are observed in the RPE as well as the basal vitread surface of the neural retina. (**E-H**) *TFAP2A* is expressed in the SE and lens vesicle. Results using sense control probes are shown in (**B,D,F,H**). il, inner layer of the optic cup; le, lens vesicle; nr, neural retina; ol, outer layer of the optic cup; rpe, developing RPE. Scale bars: 50 μm (**A,B,E,F**), 100 μm (**C,D,G,H**), 25μm (**C′**).

Figure S10

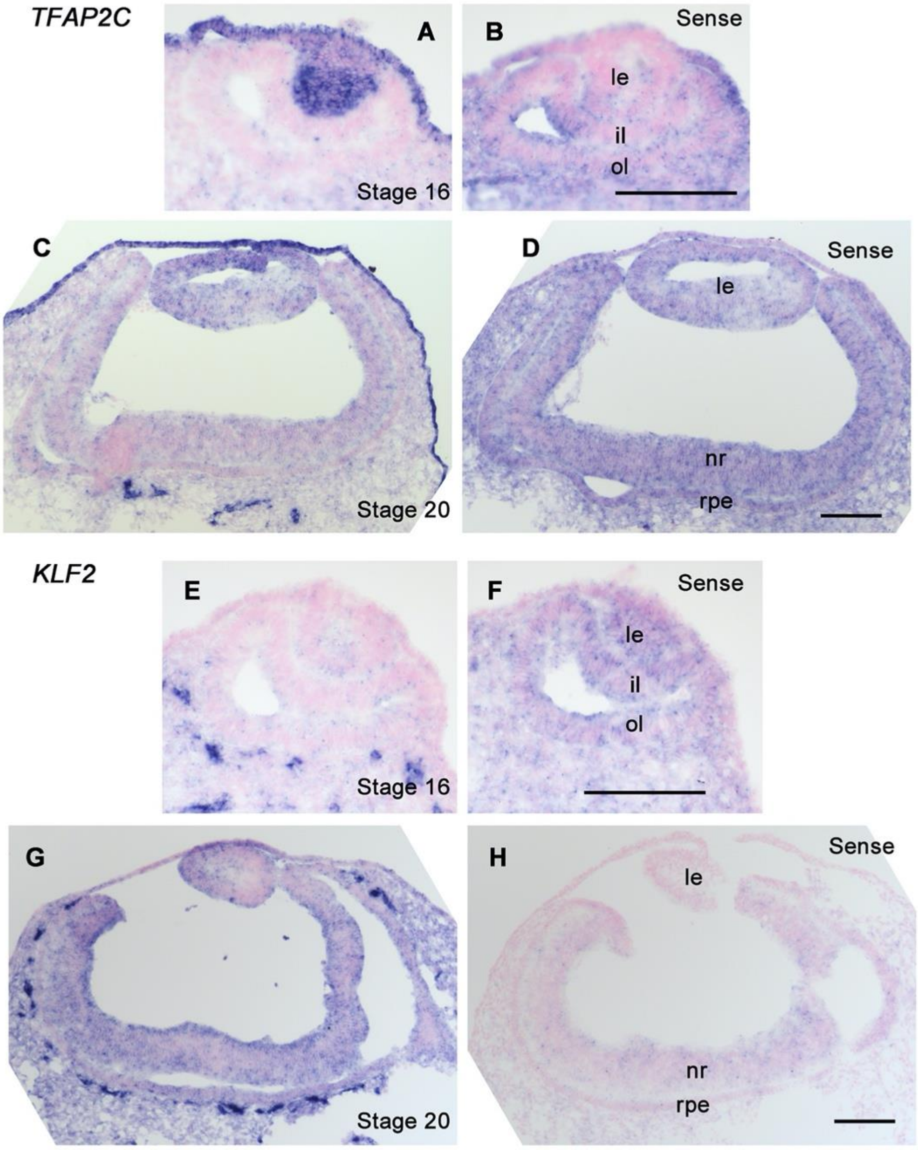

Figure S10: Expression patterns of *TFAP2C* and *KLF2* in the developing chicken eye. (**A-D**) *TFAP2C* is expressed in the surface ectoderm (SE), lens vesicle, and by a subset of cells in the periocular mesenchyme (**C**). (**E-H**) *KLF2* is expressed by a subset of cells in the periocular mesenchyme just beneath the RPE (**G**). il, inner layer of the optic cup; le, lens vesicle; nr, neural retina; ol, outer layer of the optic cup; rpe, developing RPE. Scale bars: 100 μm.

Figure S11

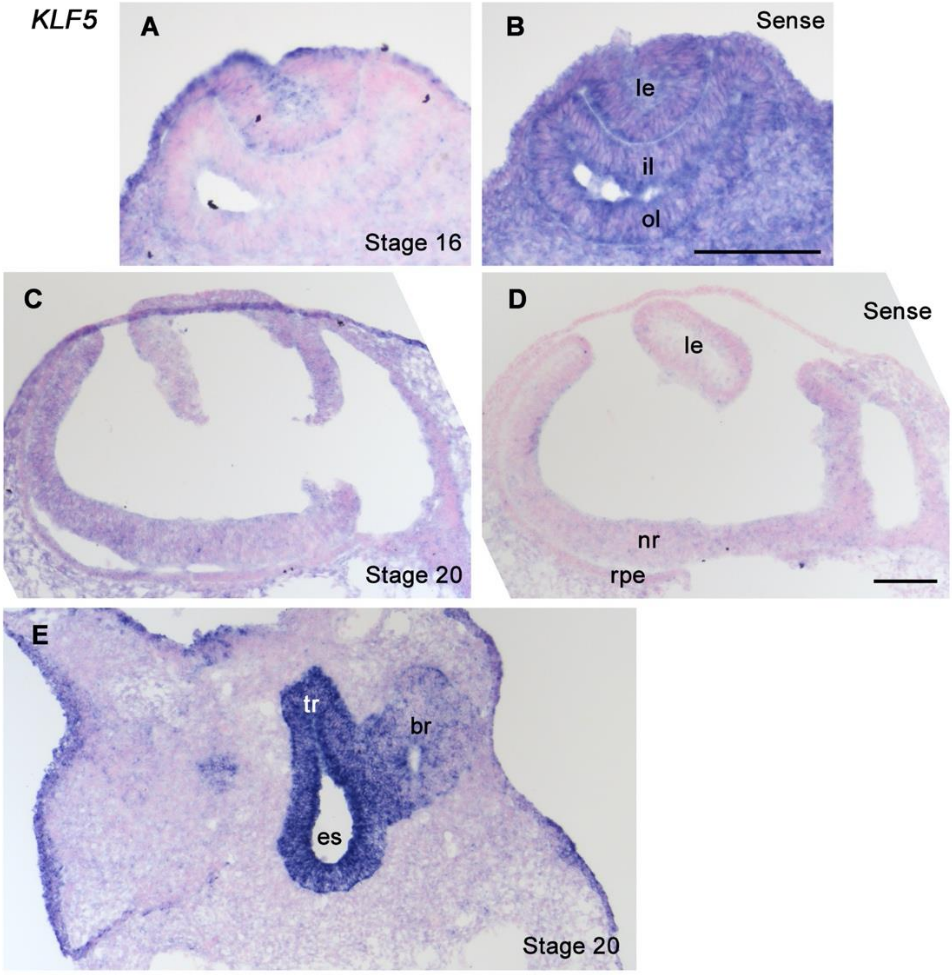

Figure S11: Expression pattern of *KLF5* in the developing chicken eye. *KLF5* is expressed in the surface ectoderm of the developing eye (**A,C**) and outside the eye (**E**) as well as the endoderm of developing trachea, bronchus and esophagus. br, bronchus; es, esophagus; il, inner layer of the optic cup; le, lens vesicle; nr, neural retina; ol, outer layer of the optic cup; rpe, developing RPE; tr, trachea. Scale bars: 100 μm.

Table S1: PCR primer sequences used in this study.

Gene name	Amplicon size (bp)	Primer name	Primer/DNA sequences (5'->3')	Purpose
chicken BHLHE40 (cBHLHE40)	1193	cBhlhe40 forward	CTGACCCTGAAGCACGTGAA	cloning a template cDNA to synthesize riboprobes
		cBhlhe40 reverse	GACACATGGCAGCTGACTGA	
cBHLHE40	1206	cBhlhe40 full forward	ATGGAGCGCATCCCCAGC	cloning a full-coding sequence for *cBHLHE40*
		cBhlhe40 full reverse	TTAGTCTTTGGTTTCCAAGTTCAAC	
cBHLHE40	1252	Bhlhe40_Fw	TAGCGTTTAAACTTAAGCTTGCCACCATGGAGCGCATCCCCAGC	insertion of *cBHLHE40* into pCAGGS vector
		Bhlhe40_Rv	ACGGGCCCTCTAGACTCGAGTTAGTCTTTGGTTTCCAAGTTCAAC	
cBHLHE40	1279	Bhlhe40_pCAG_Fw	AGCGTTTAAACTTAAGCTTGGCCACCATGGAGCGCATC	insertion of *cBHLHE40* into pCAGGS vector with HA-tag
		Bhlhe40_HA_pCAG_Rv	CGGGCCCTCTAGACTCGAGCTTACGCATAATCCGGCACATCATACGGATAGTCTTTGGTTTCCAAGTT	
delta_basic cBHLHE40	194	ggaBhlhe40_delbasic_Fw	AGGAGCGAGGACAGCAAGGATAGGATCAACGAGTGC	amplification of N terminal portion of HA-tagged cBHLHE40 before basic region
		ggaBhlhe40_delbasic _Rv	GCACTCGTTGATCCTATCCTTGCTGTCCTCGCTCCT	
delta_basic cBHLHE40	1076	pCAG_fw_HindIII	AGCGTTTAAACTTAAGCTTG	amplification of C terminal portion of HA-tagged cBHLHE40 after basic region
		pCAG_Rv_XhoI	CGGGCCCTCTAGACTCGAGC	
acidic extension cBhlhe40	106	a-Bhlhe40_ins_Fw_pCAG	AGCGTTTAAACTTAAGCTTGGCCACCATGGCATCAATGACTGGA	acidic extension sequence for replacement of N terminal portion of cBHLHE40 including basic region
		a-Bhlhe40_ins_Rv	AACTCTTCCAGCTCTTCTTCATCATCCTCCTCCTCATCGGGGTCCCTCCCCATCTGCTGACCTCCAGTCATTGATGCCAT	
acidic extension cBhlhe40	1068	a-Bhlhe40_Fw	AGAAGAGCTGGAAGAGTTGGAAGAGTGCATCGCCCAGCTC	amplification of C terminal portion of HA-tagged cBHLHE40 after basic region
		pCAG_Rv_XhoI	CGGGCCCTCTAGACTCGAGC	
chicken BHLHE41	1776	gga_bhlhe41_Fw	CCCGCGGATCCCCGAAGC	cloning template cDNAs to synthesize riboprobes
		gga_bhlhe41_Rv	GCACTGCGGCCGCCCATAAA	
chicken TEAD3	1299	gga_tead3_Fw	ATGGACAAGAGCCTGGACAATGACGCCGAG	
		gga_tead3_Rv	CTAGTCCTTCACCAACTTGTAGACATGATG	
chicken TFAP2A	1640	gga_tfap2a_Fw	GGGATCGGACCCTCTCCCGC	
		gga_tfap2a_Rv	CTGGCTCCGTCCCCCTGTTT	
chicken TFAP2C	1347	gga_tfap2c_Fw	ATGTTGTGGAAACTGGCAGATAACGTCAAG	
		gga_tfap2c_Rv	TTACTTCCTGTGCTTGTCCATTTTATCGAT	
chicken KLF2	1536	gga_klf2_Fw	CCATGGCGCTGAGCGATACCA	
		gga_klf2_Rv	AACACCGAGCTACAGCAACGCTC	
chicken KLF5	1504	gga_klf5_Fw	GAGGCATTGACGACGCCCGA	
		gga_klf5_Rv	GCCTCGGCAACAACTCTGGCT	

Table S2: Antibodies used in this study.

Antibody		Vender	Catalog#	Dilution	Final concentration	Diluted in	Antigen retrieval	Antibody concentration
Anti-OTX2	Rabbit polyclonal	abcam	ab21990	1000	1 ug/mL	IMMUNO SHOT immunostaining, Fine	None	1 mg/mL
Anti-VSX2	Sheep polyclonal	Ex Alpha	X1180p	400	0.25 ug/mL	IMMUNO SHOT immunostaining, Mild	in TE (pH 8.0) at 98°C, 45 min	1 mg/mL
Anti-Cleaved caspase 3	Rabbit monoclonal	R & D systems	MAB835	1000	0.5 ug/mL	IMMUNO SHOT immunostaining, Fine	None	0.5 mg/mL
Anti-HA	Rat monoclonal	Roche	ROAHAHA	200 or 400	0.5 or 0.25 ug/mL	IMMUNO SHOT immunostaining, Fine or Mild	None	0.1 mg/mL
Anti-OTX2 + Anti-HA				1000(OTX2), 200 (HA)		IMMUNO SHOT immunostaining, Fine	None	
Anti-VSX2 + Anti-HA				400(VSX2), 400 (HA)		IMMUNO SHOT immunostaining, Mild	in TE (pH 8.0) at 98°C, 45 min	
Anti-sheep IgG Alexa Fluor 488	Donkey polyclonal	Thermo Fisher Scientific	A-11015	1000	2 ug/mL	1% BSA, 0.1% Triton-X100 in PBS	Not applicable	2 mg/mL
Anti-rat IgG Alexa Fluor 488	Goat polyclonal	Thermo Fisher Scientific	A-11006	1000	2 ug/mL	1% BSA, 0.1% Triton-X100 in PBS		2 mg/mL
Anti-rat IgG Alexa Fluor 568	Donkey polyclonal	abcam	ab175708	1000	2 ug/mL	1% BSA, 0.1% Triton-X100 in PBS	Not applicable	2 mg/mL
Anti-rabbit IgG Alexa Fluor 647	Goat polyclonal	abcam	ab150087	1000	2 ug/mL	1% BSA, 0.1% Triton-X100 in PBS	Not applicable	2 mg/mL
